# Single-End Adaptive Optics Compensation for Emulated Turbulence in a Bi-Directional 10-Mbit/s per Channel Free-Space Quantum Communication Link Using Orbital-Angular-Momentum Encoding

**DOI:** 10.34133/2019/8326701

**Published:** 2019-02-24

**Authors:** Cong Liu, Kai Pang, Zhe Zhao, Peicheng Liao, Runzhou Zhang, Haoqian Song, Yinwen Cao, Jing Du, Long Li, Hao Song, Yongxiong Ren, Guodong Xie, Yifan Zhao, Jiapeng Zhao, Seyed M. H. Rafsanjani, Ari N. Willner, Jeffrey H. Shapiro, Robert W. Boyd, Moshe Tur, Alan E. Willner

**Affiliations:** ^1^Department of Electrical Engineering, University of Southern California, Los Angeles, CA 90089, USA; ^2^Department of Physics and Astronomy, University of Southern California, Los Angeles, CA 90089, USA; ^3^The Institute of Optics, University of Rochester, Rochester, New York 14627, USA; ^4^Massachusetts Institute of Technology, Research Laboratory of Electronics, Cambridge, Massachusetts 02139, USA; ^5^School of Electrical Engineering, Tel Aviv University, Ramat Aviv 69978, Israel

## Abstract

A single-end adaptive-optics (AO) module is experimentally demonstrated to mitigate the emulated atmospheric turbulence effects in a bi-directional quantum communication link, which employs orbital angular momentum (OAM) for data encoding. A classical Gaussian beam is used as a probe to detect the turbulence-induced wavefront distortion in the forward direction of the link. Based on the detected wavefront distortion, an AO system located on one end of the link is used to simultaneously compensate for the forward and backward channels. Specifically, with emulated turbulence and when the probe is turned on, the mode purity of photons carrying OAM *ℓ* = 1 is improved by ~ 21 % with AO mitigation. We also measured the performance when encoding data using OAM {*ℓ* = −1, + 2} and {*ℓ* = −2, + 1} in the forward and backward channels, respectively, at 10 Mbit/s per channel with one photon per pulse on average. For this case, we found that the AO system could reduce the turbulence effects increased quantum-symbol-error-rate (QSER) by ~ 76 % and ~ 74 %, for both channels in the uni-directional and bi-directional cases, respectively. Similar QSER improvement is observed for the opposite direction channels in the bi-directional case.

## 1. Introduction

Quantum optical communications holds the promise for secure transfer of data over both free-space and fiber links [1–7]. Commonly, data could be encoded on two orthogonal quantum states of the photon, e.g., polarization [2–4]. Orthogonality between the states is important in order to limit any crosstalk from one state onto the other when encoding, transmitting, and decoding the single photon.

Importantly, there has been interest in quantum systems that utilize a larger number of orthogonal states by using a larger “alphabet” set of characteristics of photons. Such a larger alphabet might enable better performance in quantum communication links, such as: (i) a larger transmission capacity in terms of bits/sec, since an alphabet of 2^N^ possible values can transmit N bits per symbol period; and (ii) a higher photon efficiency in terms of bits/photon, since a single photon can now carry N bits of information instead of 1; this is very similar to the difference between binary data encoding using {0, 1} and M-ary data encoding using {0, 1,…, M-1} [8, 9].

One possible approach to a larger quantum alphabet could be utilizing a spatial modal basis set for which a single photon could occupy one of many different orthogonal spatial modes [5–7, 9–14]. One possible basis set is orbital-angular-momentum (OAM) modes, which is a subset of circularly symmetric Laguerre-Gaussian (LG) modes [15, 16]. OAM-carrying photons have phasefronts that “twist” at different rates and are orthogonal to each other when propagating coaxially. The OAM-carrying photon also exhibits a vortex, ring-like accumulated intensity profile with little power in the center [10, 15, 16].

In a uni-directional quantum free-space optical (FSO) communications link using OAM encoding, the transmitter would encode each photon on one of many possible OAM states and transmit the photon through free space and the receiver would detect its OAM state [6]. For higher performance, it is often considered advantageous to enable bi-directional data transmission in a quantum communication system [17].

In general, a key limitation of both classical and quantum FSO links is the system performance degradation due to atmospheric turbulence [18–23]. For example, turbulence affects the wavefront of a photon, such that the unique spatial phase profile that defines its OAM state would be distorted. Turbulence is perhaps more challenging for OAM encoding of a single photon than for polarization encoding, and turbulence can decrease OAM modal purity and thereby increase intermodal crosstalk [18, 21, 22]. Adaptive optics (AO) have been shown to help mitigate turbulence in: (i) single-directional OAM-based quantum links [23, 24] and (ii) bi-directional OAM-based classical links [25]. To our knowledge, there has been little reported work on using AO in bi-directional quantum links, especially for OAM-encoded links.

In this paper, we demonstrate using single-end AO compensation for emulated turbulence in an OAM-encoded, bi-directional FSO quantum communication link at 10 Mbit/s per channel. A rotatable phase screen plate with pseudo-random phase distributions obeying Kolmogorov spectrum statistics is used to emulate turbulence effects in the laboratory environment [18, 24]. Specifically, with emulated turbulence and when the probe is turned on, the mode purity of photons carrying OAM *ℓ* = 1 is improved by ~ 21 % with AO mitigation. As a proof-of-concept experiment, data is encoded with OAM values of {*ℓ* = −1, +2} and {*ℓ* = −2, +1} for the forward and backward channels, respectively. For this case, we found that the AO system could reduce the turbulence effects increased quantum-symbol-error-rate (QSER) by ~ 76 % and ~ 74 %, for both channels in the uni-directional and bi-directional cases, respectively.

## 2. Background

### 2.1. Introduction to OAM

A light beam carrying OAM has a helical phasefront and its wave vector spirals around the beam axis. The phase term of an OAM beam is described as exp⁡(*iℓθ*) in the transverse plane, where *θ* refers to the azimuthal coordinate and *ℓ* defines the “charge” carried by the OAM mode. *ℓ* describes the number of 2*π* phase shifts occurring in the azimuthal direction and could be a positive, negative, or a zero-value integer, corresponding to clockwise, counterclockwise phase helices, or a conventional non-OAM Gaussian beam with power in the beam center, respectively [15, 16, 26].

An OAM beam can be generated at the transmitter by inverting a Gaussian beam into a certain OAM-carrying beam. For example, this could be achieved by using spiral phase plates [16] or phase holograms [27, 28]. To detect an OAM beam at the receiver, an OAM beam can be converted back to a Gaussian-like beam by passing it through a phase distribution that is a conjugate of the transmitter.

### 2.2. OAM for Communications

The orthogonality of OAM modes can be utilized for communications: (i) by multiplexing, in which multiple independent data-carrying beams each having a different OAM value can be transmitted simultaneously; (ii) by encoding, in which each data symbol is represented by one of many different OAM values.

OAM multiplexing could be beneficial for increasing the capacity in communication links [26, 29]. Since each OAM-carrying beam has an independent data stream, the total link capacity could be multiplied by the number of transmitted beams. Orthogonality of the beams could enable efficient multiplexing at the transmitter, co-propagation of overlapping beams, and demultiplexing of the beams at the receiver with inherently low crosstalk.

OAM-based data encoding could also be beneficial for increasing efficiency in a communication link. N different data symbols of {0, 1,…, N-1} could be encoded onto N different OAM states, thus increasing the photon efficiency of the communication link. OAM data encoding could be implemented using: (a) a tunable OAM mode converter with time-varying phase patterns [5], or (b) multiple fixed OAM mode converters together with an optical switch, such that the beam is switched among the different converters to carry time-varying OAM states [8]. OAM encoding could be used for either classical or quantum communication links [8, 9].

### 2.3. Quantum Communications Using OAM

Optical quantum communications allow for the transmission of quantum information between sending and receiving parties at the single-photon level, and has the ability to protect the transmitted data against eavesdropping [1–7, 10]. Commonly, quantum communications utilizes the two orthogonal polarization states of a photon for data encoding, in which one bit/photon of information is transmitted [2–4].

We think that OAM modes have the potential to be used as qubits in various quantum key distribution (QKD) schemes, such as the BB84 protocol [5–7, 9, 10]. A single photon can occupy a distinct OAM state (*ℓ*) from many available OAM states, which makes it possible to encode multiple bits of information onto a single photon [5, 9]. An N-OAM-encoded single photon could carry log_2_⁡*N* bits/photon of information, thereby potentially achieving higher photon efficiency in the system. We note that the state-of-the-art photon efficiency and secure key rate, to the best of our knowledge, of an OAM-encoding based QKD system were reported in [5], where 2.05 bits per sifted photon and 6.5 bits/second were demonstrated, respectively.

### 2.4. Atmospheric Turbulence and AO Compensation to OAM-Based Links

The inhomogeneity in the temperature and pressure of the atmosphere can lead to variations of the refractive index in the transmission path of an FSO link. The variations of refractive index can introduce phase distortion to the helical phasefront of an OAM-carrying beam or photon, thereby increasing modal coupling and inter-modal crosstalk [18, 21, 22].

An AO system can be used to mitigate the turbulence effects for both non-OAM and OAM-based FSO links. In a typical AO system, the wavefront distortion of a beam could be measured by a wavefront sensor (WFS). Based on this measurement, an error correction pattern could be derived and sent to a wavefront corrector (e.g., a spatial light modulator (SLM) or a deformable mirror) through a feed-back loop [24]. Due to the phase structure of an OAM beam, it would be challenging to directly measure its phasefront using typical Shack-Hartmann WFSs. This might happen because there is a singular point at the center of the OAM beam, which could cause inaccuracy when measuring the phasefront using typical Shack-Hartmann WFSs. One approach could be using a Gaussian probe beam on a different polarization or wavelength that can be easily separated out. This Gaussian probe can be measured by a Shack-Hartmann WFS and be readily for being separated out [9, 24].

### 2.5. Uni-Directional and Bi-Directional OAM-Based FSO Links

Bi-directional quantum links could be helpful in the following situations: (a) two independent quantum channels in different directions are desired, and (b) there are some quantum protocols that might need the bi-directional link [30, 31]. Moreover, in a uni-directional OAM-based FSO link propagating through turbulent atmosphere, a transmitter, receiver, and AO system would likely be required. To achieve full-duplex bi-directional data transmission, one could use a receiver and transmitter at each end as well as an AO system at each end. Alternatively, a bi-directional link could use only one AO system located at one end of the link [17].

The reciprocity of atmospheric turbulence suggests that the coaxially counter-propagating beams through the same turbulence medium may experience similar turbulence distortions [32, 33]. This implies that in a bi-directional OAM-encoded quantum link, an AO system that corrects distorted OAM-carrying photons for one direction, could also compensate for the opposite direction. In the previous study, a single-end AO module has been demonstrated for simultaneous pre- and post-compensation for a bi-directional OAM-multiplexed classical FSO link [25]. In this paper, we utilize a similar compensation method in the quantum domain, as was shown in [25] for the classical domain. Our differences from previous classical-domain works are as follows: (1) we use attenuated laser sources (or SPDs) to generate (or receive) either a single photon or a few photons which might be more sensitive to noise compared with the classical-domain case [5, 9], (2) we explore the use of AO compensation to reshape the probability distribution of a single photon (or a few photons) on different OAM modes, whereas the classical-domain case focuses on the power distribution, and (3) we measure the quantum channels in the existence of the classical probe beam.

## 3. Concept

**Figure 1:**
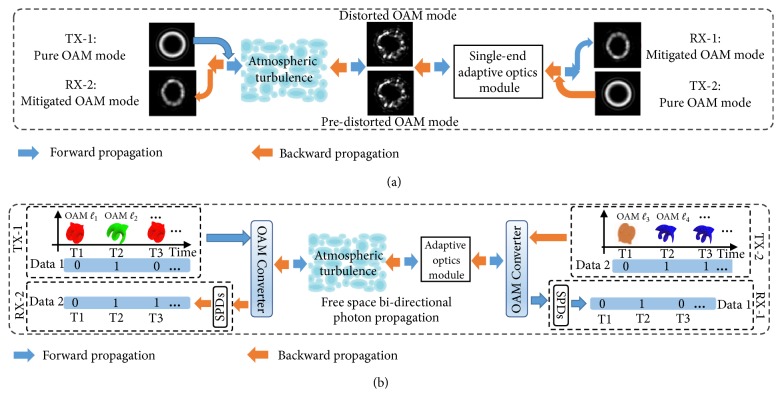
Concept diagram of (a) using a single-end AO module for the bi-directional coaxis OAM mode propagation through atmospheric turbulence and (b) simultaneous pre- and post-AO compensation for a turbulence-induced, OAM-encoded, and bi-directional quantum communication link. TX-1/2: transmitter for the forward/backward quantum channel; RX-1/2: receiver for the forward/backward quantum channel; SPD: single photon detector.

Figure 1(a) depicts the concept of counter-propagating OAM modes through atmospheric turbulence with single-end AO compensation. Two groups of OAM-carrying photons coaxially propagate through the atmosphere in opposite directions. The AO module placed at one end of the link (TX-2 side) is used to compensate the phase distortion of the received OAM photons from TX-1 as the post-compensation. The wavefront of the OAM-carrying photons coming from TX-2 are modulated by the wavefront corrector, with the same correction pattern used in the post-compensation, in the AO module as the pre-compensation. These photons then propagate backward through the same turbulent atmosphere. Figure 1(b) shows the concept diagram of simultaneous pre- and post-AO turbulence mitigation for an OAM-encoded, bi-directional quantum communication link transmitting through the atmosphere. The forward and backward data-carrying quantum channels are each encoded with two different sets of OAM modes, generated at TX-1 and TX-2, respectively. The phasefronts of the transmitted OAM photons will be distorted by the turbulence effects, and the AO module could potentially reduce the distortion-induced degradation for both the forward and backward channels simultaneously [25, 35].

## 4. Experimental Setup

**Figure 2:**
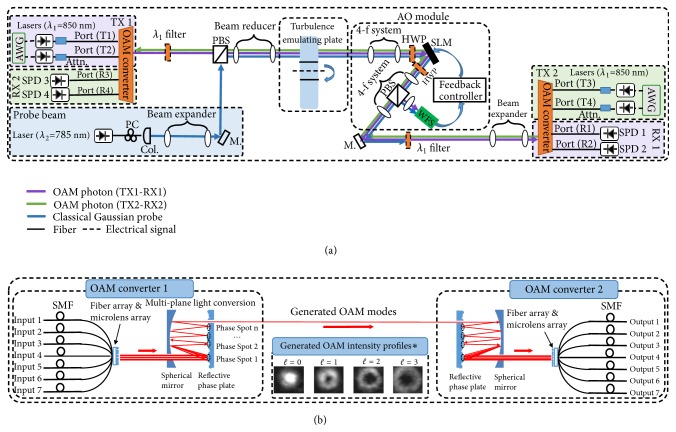
(a) Experimental setup of the AO-compensated, bi-directional, and OAM-encoded quantum communication link. (b) The OAM converter pairs used in the setup and the generated OAM intensity profiles [34]. AWG: arbitrary waveform generator; Attn.: attenuator; PBS: polarizing beam splitter; Col.: collimator; PC: polarization controller; HWP: half-wave plate; SLM: spatial light modulator; WFS: wavefront sensor; SPD: single photon detector; M.: mirror. *∗*The OAM intensity profiles are collected by using charge coupled device- (CCD-) based camera in the classical domain.

Figure 2(a) presents the experimental setup. Two quantum channels are generated at TX-1 and TX-2 by directly modulated lasers at *λ*_1_ = 850 nm. Fiber-based attenuators are used to attenuate the power of data channels to a single-photon level. After the attenuation, custom-designed, fiber-input, free-space-output multi-plane light conversion- (MPLC-) based OAM converters (shown in Figure 2(b)) transform Gaussian photons into different OAM photons. Each converter has 7 single-mode fiber (SMF) inputs corresponding to OAM {*ℓ* = −3, − 2, − 1, 0, + 1, + 2, + 3}. In the converter, the SMFs are connected to a fiber array followed by a microlens array, collimating the Gaussian photons to propagate in free space. The free-space Gaussian photons are sent to a multi-pass cavity where the photons are reflected 15 times at different locations on a reflective phase plate. In each reflection, the wavefront of the photons are shaped by different transverse phase profiles. The succession of these transverse phase profiles forms a spatial unitary transform that converts the Gaussian photons to coaxially propagating OAM photons with different orders (*ℓ*) [34].

A classical Gaussian probe beam at *λ*_2_ = 785 nm placed at the TX-1 side is orthogonally polarized with respect to the quantum channels. The probe is expanded to the same diameter of the generated OAM beam with the largest beam size (i.e., *ℓ* = −3 beam in this experiment), which is 5.2 mm. The expanded Gaussian beam is then combined with the quantum channels by using a polarizing beam splitter (PBS). A 1.5:1 beam reducer is used to adjust the combined beam size such that the beam could be fully captured by the SLM and the WFS. The reduced probe beam diameter (D) is 3.24 mm. The combined probe beam and quantum channels coaxially propagate through the turbulence emulator and the AO module. The turbulence emulator is characterized by its Fried parameter, *r*_0_ = 1 mm [18]. The ratio *D*/*r*_0_ can be used to describe the turbulence strength (*D*/*r*_0_ = 3.24 in this experiment) [18]. The AO module is placed at the TX-2 side, to mitigate distorted OAM photons from TX-1 as the post-compensation. In the AO module, the probe beam serves for wavefront distortion estimations and correction pattern retrieval [23–25]. The SLM serves as a wavefront corrector. Two half-wave plates (HWPs) are used to align the linear polarizations of the coming beams to the orientation of the SLM. The WFS detects the wavefront distortion of the probe and the SLM is imaged with the WFS using a 4-*f* lens system. A feedback loop is used to send the correction pattern derived from the WFS to the SLM. The total length of the free-space optical path is ~1.5 m.

The mitigated quantum channel from TX-1 passes through another PBS and a free-space bandpass filter centered at *λ*_1_ to filter out the probe beam. The PBS provides ~30 dB isolation in polarization, and the *λ*_1_-filter provides ~70 dB isolation in wavelength. The OAM photons are enlarged by a 1:1.5 beam expander and then counter-propagate through another MPLC-based OAM converter. By using the OAM converter reversely, OAM photons could be transformed back to Gaussian photons which are then sorted to corresponding SMF outputs. The demultiplexed photons are then detected at RX-1 using silicon avalanche photodiode- (APD-) based single-photon detectors (SPDs). The SPDs have 50 ns deadtimes, 0.5 % after-pulsing probabilities, 500 count/sec dark count rates, and ~ 40 % quantum efficiencies at the wavelength of 850 nm. The SPDs have quantum efficiencies of ~50 % at 785nm which is the wavelength of our classical Gaussian probe beam. They also have ~40-60 % quantum efficiencies at a wavelength range of 500-850 nm (other wavelengths have lower quantum efficiencies). To minimize the effects of the surrounding light, we place our setup in a darkroom and use free-space bandpass filters of 850±10 nm wavelength range at the two sides of the quantum link. The detected events are recorded for offline digital signal processing [9]. We observe that the quantum link is relatively stable and the measured data points do not fluctuate much. We note that only a single measurement is taken for every data point. The statistical errors could be added if multiple measurements are taken for a single data point. The quantum channel transmitted from TX-2 is first sent to the AO module and then propagated through the turbulence emulator. Consequently, this quantum channel is pre-distorted by the correction patterns on the SLM in the AO module as the pre-compensation, before experiencing the turbulence distortion. Finally, this quantum channel is received by RX-2 for demultiplexing and detection.

## 5. Experimental Results

### 5.1. AO Compensation for OAM-Carrying Photons in Quantum Communication Systems

**Figure 3:**
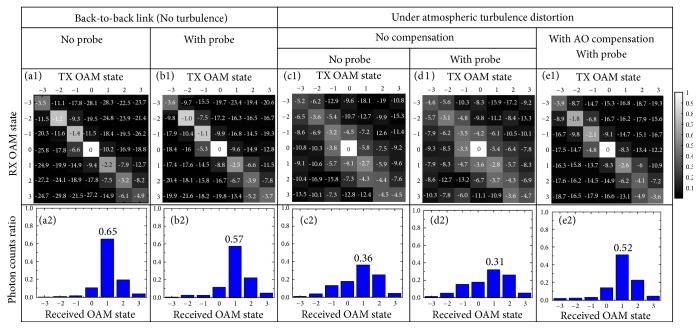
(a1-e1) Channel transfer matrices when sending OAM modes {*ℓ* = −3,…, +3}, respectively, for different cases. The numbers in (a1-e1) are measured in the quantum domain as the ratio of the measured photon counts to the maximum photon counts in this matrix in a unit of dB. (a2-e2) The photon counts ratio on received OAM modes {*ℓ* = −3,…, +3} when sending only OAM *ℓ* = 1 photons for different cases. TX: transmitter; RX: receiver.

Figures 3(a1)-3(e1) show the quantum channel transfer matrices: (i) in the back-to-back link (without the turbulence emulator in the optical path) with the probe being turned off (Figure 3(a1)) and on (Figure 3(b1)), (ii) under a random turbulence realization with the probe off (Figure 3(c1)) and on (Figure 3(d1)) without AO mitigation, and (iii) with AO mitigation while the probe is on (Figure 3(e1)). Only the forward quantum channel is transmitted in the link and the power of the classical probe is ~  72  dB higher than that of the quantum channel. In the back-to-back link (Figure 3(a1)), when sending OAM *ℓ* photons, most of the received photons remain in the *ℓ*-th OAM order because of the orthogonality between OAM modes. The influence of the classical probe on the quantum channel is shown in Figure 3(b1). Turbulence effects could induce the distortion to the wavefront of OAM-carrying photons and increase the probability of the OAM photons existing in the undesired orders (Figure 3(c1)). The influence of the probe on the disturbed quantum channel is shown in Figure 3(d1). With AO mitigation, photons are better confined to their desired OAM orders, as shown in Figure 3(e1). Figures 3(a2)-3(e2) show the photon leakage from the OAM *ℓ* = 1 mode to the other modes when only OAM *ℓ* = 1 photons are sent. In the back-to-back link with the probe off (Figure 3(a2)), 65 % of the received photons would stay in the desired mode. We note that this nonunity of the received OAM *ℓ* = 1 photon ratio might be due to the nonideal performance of optical components (e.g., OAM converters), the imperfection of the optical link alignment, and the noise at the SPDs. The received OAM *ℓ* = 1 photon count ratio reduces to 57 % because of the power leakage of the probe (Figure 3(b2)). With turbulence effects, this percentage decreases to 36 % with the probe off (Figure 3(c2)) and 31 % with the probe on (Figure 3(d2)). This is improved by the AO mitigation: 52 % of the received photons remain in the desired mode with the AO mitigation (Figure 3(e2)). The mode purity of OAM *ℓ* = 1 photons is improved by ~ 21 % with the AO mitigation when the probe is on.

**Figure 4:**
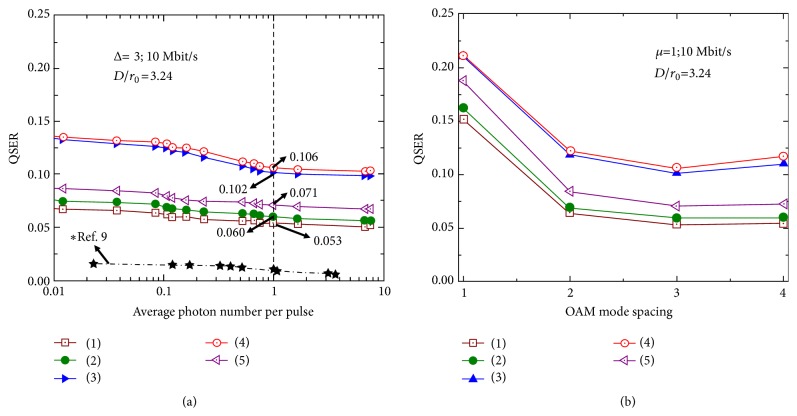
QSER for (a) the OAM {*ℓ* = −2, +1}-encoded quantum link as a function of the average photon number per pulse (*μ*) and (b) the two-OAM-encoded link as a function of OAM mode spacing when *μ* = 1. Different cases: back-to-back link (1) with no probe beam and (2) with probe beam; under the influence of turbulence effects (3) without any compensation without the probe beam, (4) without any compensation with the probe beam, and (5) with AO compensation with the probe beam. QSER: the ratio of accumulated error symbols (photons) to the total registered symbols (photons). *∗*The dash-dot lines are experimental results from [9], in which the encoding OAM modes are {*ℓ* = +1, +4} generated and received by SLMs.

Figure 4(a) shows the QSER curves as functions of the average photon number per pulse (*μ*) under the five different situations in Figure 3. The QSER is defined as the ratio of accumulated error symbols (photons) to the total registered symbols (photons). Only the forward channel is transmitted at 10 Mbit/s and the channel is encoded with OAM {*ℓ* = −2, +1}. When *μ* = 1, the back-to-back QSER increases from 0.053 to 0.06 under the influence of the power leakage of probe beam. Under the influence of turbulence effects, the QSER increases to 0.102 with the probe off and 0.106 with the probe on. This QSER is reduced to 0.071 with AO mitigation while the probe is on. The AO mitigation reduces ~ 76 % ((0.106 − 0.071)/(0.106 − 0.060) × 100  %) of the additional QSER increased by the turbulence effects. We note that in [9], the QSER for the back-to-back link is < 0.02 when *μ* = 1; this might be because in [9] the SLMs are used to generate and receive encoding OAM-carrying photons of {*ℓ* = +1, +4}, which have < -28 dB intermodal crosstalk. Figure 4(b) shows the AO compensation for the quantum channel encoded with OAM modes with different mode spacings. The channel is transmitted at 10 Mbit/s when *μ* = 1. We use OAM mode groups {*ℓ* = −1, −2}, {*ℓ* = −1, +1}, {*ℓ* = −2, +1}, and {*ℓ* = −2, +2} for the OAM mode spacing ∆ = 1, 2, 3, 4, respectively. The maximum back-to-back intermodal quantum channel crosstalk values for these mode spacings are -9.3 dB, -14.9 dB, -19.9 dB, and -23.9 dB with the probe off, respectively. We can see from Figure 4(b) that, for a fixed *μ*, smaller OAM mode spacing tends to result in larger back-to-back QSERs due to the crosstalk effects. The AO mitigation tends to provide a better QSER improvement when the back-to-back QSER is smaller, which correspond to a larger OAM mode spacing. When ∆ > 2 the channel crosstalk is <−15 dB, and the QSERs tends to decrease slower with the increase of the OAM mode spacing.

### 5.2. AO Compensation for a Two-OAM Encoded, Bi-Directional Quantum Communication Link

**Figure 5:**
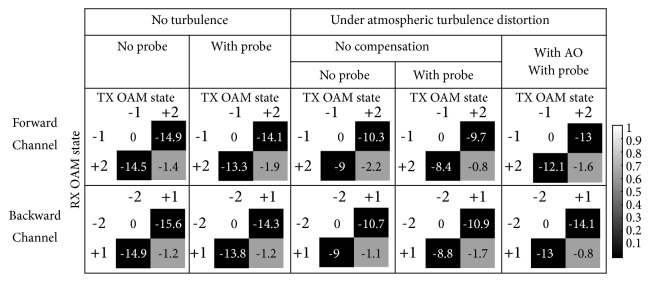
The crosstalk matrices between encoded OAM states measured in the quantum domain under different situations in a bi-directional quantum communication link. The numbers in the matrices are normalized and in a unit of dB. The forward and backward channels are encoded by OAM {*ℓ* = −1, +2} and OAM {*ℓ* = −2, +1}, respectively. TX: transmitter; RX: receiver.

Figure 5 shows the crosstalk matrices between encoded OAM modes in the bi-directional quantum link under the following situations (left to right): the back-to-back link with the probe turned off and on, under a random turbulence realization without AO compensation when the probe is turned off and on, and under the same turbulence realization with the AO compensation when the probe is on. The forward and backward channels are encoded with OAM {*ℓ* = −1, +2} and OAM {*ℓ* = −2, +1}, respectively. The power of the classical probe is ~ 72 dB higher than that of each quantum channel. In the back-to-back link, the orthogonality between different OAM modes provides a ~ -14 dB crosstalk between encoding OAM modes. The crosstalk of the bi-directional case is slightly higher than that of the single-directional case for the same encoding OAM modes. This is perhaps majorly due to the power reflection in the OAM converters. In the bi-directional case, the turbulence effects added ~ 5 dB of crosstalk, which could be reduced by ~ 4 dB with AO mitigation.

**Figure 6:**
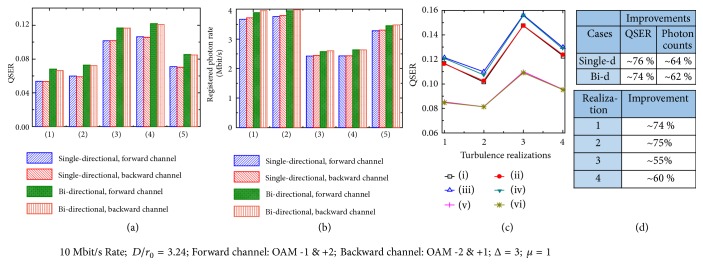
(a) The QSER and (b) the registered photon rate in single-directional and bi-directional quantum links for one random turbulence realization. Different cases in (a-b): back-to-back link (1) with no probe beam and (2) with the probe beam; under the influence of turbulence effects (3) without compensation without the probe beam, (4) without compensation with the probe beam, and (5) with AO compensation with the probe beam. (c) QSERs under four different turbulence realizations. Different cases in (c) are as follows: (i) and (ii) are forward and backward channels without compensation without the probe beam; (iii) and (iv) are forward and backward channels without compensation with the probe beam; (v) and (vi) are forward and backward channels with AO compensation with the probe beam, respectively. The turbulence strength of D/r_0_ = 3.24 is applied in (a-c). (d) The improvements of the AO mitigation in (a-c).

Figure 6 shows the QSER (Figure 6(a)) and the registered photon rate (Figure 6(b)) of the single-directional and bi-directional quantum communication links at 10 Mbit/s for each direction under one random turbulence realization. Figure 6(c) presents the QSERs under four different turbulence realizations. Figure 6(d) shows the AO mitigation improvements for Figures 6(a)–6(c). The quantum link is encoded by OAM mode groups {*ℓ* = −1, +2} and {*ℓ* = −2, +1} for the forward and the backward quantum channels, respectively. It could be seen from Figure 6(a) that the AO system could help to reduce the turbulence-induced QSER by ~76 % and ~74 % and improve the registered photon rates by ~64 % and ~62 %, for the single-directional and the bi-directional link, respectively. Photon's wavefront is distorted because of the turbulence effect, and this increases the probability of the OAM photons to be detected in the undesired orders, causing a larger QSER measurement. The higher error rate could be mitigated by the AO system. The turbulence effects also cause less photon registration, perhaps because the photons are distorted and cannot be demultiplexed to *ℓ* = 0 photons and recorded by the SPDs. This reduction could also be mitigated by the AO system. We also notice that the improvements in the forward and backward channels are similar (the difference is < 1 %) for the same case. This similar improvement might be due to the similar crosstalk in the opposite directions. Figure 6(c) shows that, under the four random-chose turbulence realizations, QSERs all improved by >50 % with the AO mitigation. The QSERs of the forward and backward channels for these four turbulence realizations are all similar, which also indicates the reciprocity of atmospheric turbulence and the symmetry of the AO system [32].

## 6. Conclusion and Discussion 

The demonstration of an ~74 % turbulence-induced QSER mitigation using AO compensation in an OAM-encoded, bi-directional quantum communication link explores the potential of using a single-end AO system for simultaneous pre- and post-compensation for the turbulence-distorted OAM-carrying single photon (or a few photons). There are several issues that deserve further exploration.

(i) Encoding with higher-order OAM modes might be needed to (a) achieve a better system QSER by applying a larger OAM mode spacing [9, 26] and (b) scale our approach to a higher dimension by encoding with more (>2) OAM modes, to increase the transmission capacity and the photon efficiency of a quantum link [5–7]. However, when encoding with a higher-order OAM mode, there might be: (1) a larger turbulence distortion of the OAM-carrying photons [18, 35], and (2) a larger coupling loss at the OAM converter due to the beam divergence [9, 26]. Therefore, there could be a trade-off between the encoding OAM mode spacing and the total encoding OAM mode number (N) concerning the system performance, such as the QSER. Moreover, the complexity of hardware devices could be increased when encoding with more OAM modes, for example, more phase plates might be needed for generating or receiving more OAM modes. The trade-off and the increase of hardware devices complexity could result in limiting the benefit of increasing N; thus, the photon efficiency of the quantum communication system, which is up to log_2_⁡N, might be bounded [5].

(ii) We encode OAM modes on either a single photon or a few photons, which could be considered a reasonable representation of one symbol in a quantum link, as shown in [5]. OAM encoding might be helpful in quantum QKD protocols, where one can randomly pick one set from multiple basis sets, such as an OAM basis set. The random choice of basis sets, which could provide the security of a quantum link, could be achieved with the use of a (polarizing) beam splitter, a self-designed electrical module of random number generator, or a physical thermal noise device [3–5]. We note that we do not implement the full QKD protocol. Further discussion on the OAM-based QKD protocol was reported in [5] where, for example, the security dependence of QSER was analyzed.

(iii) We emulate the single photon source by using an attenuated laser source to generate either a single photon or a few photons at a time. Besides the attenuated laser sources used in our experiment, there are quantum sources such as single photon sources and entangled photon sources, which might be beneficial for, for example, improving the security and reducing the channel loss of a quantum link [10, 36, 37]. We believe the AO configuration proposed in our work also has the ability to help with quantum links using these sources by mitigating photon distortion [37].

(iv) Other large-alphabet approaches, such as time-bin encoding, could also be implemented in FSO communication links [38]. For implementation, time-bin encoding would typically require temporal control, such as the time delay alignment, whereas OAM-encoding typically needs spatial control, such as the use of SLMs to generate OAM modes. Moreover, OAM encoding could be compatible with other approaches. Combining OAM encoding with other approaches to further increase the channel speed and security [12, 14, 29] could be a potential future research topic.

Moreover, two issues about the experimental implementation are discussed as follows.

(a) In our setup of the AO system, we use a Shack-Hartmann WFS to measure the wavefront distortion of the Gaussian probe beam. There are other alternative WFSs that could be used in AO systems for various applications, such as curvature, pyramid, and interferometer-based WFSs [39–41]. To measure the wavefront using our WFS, the power of the light should reach the minimum power requirement of the sensor inside the WFS, which is much higher than the single-photon-level power. Therefore, we use a classical probe beam for wavefront detection and then separate this probe beam using a PBS and a wavelength filter. Such separation introduces an insertion loss to the quantum channels, which might cause quantum information loss. The probe beam has photon leakage to the quantum channel, which leads to additional crosstalk. Moreover, the real-world atmospheric turbulence is dynamic, so the feedback loop in the AO system is required to have a compatible transient period with the dynamic turbulence. This could be achieved by the use of such components as fast deformable mirrors and WFSs [42].

(b) We use a single rotatable phase screen plate with a pseudo-random phase distribution obeying Kolmogorov statistics for turbulence emulation. For more accurate turbulence characterization of real-world, long-distance links, more effects should also be considered, such as the variance in turbulence conditions along the link [43].

## References

[1] Gisin N., Thew R. (2007). Quantum communication. *Nature Photonics*.

[2] Hübel H., Vanner M. R., Lederer T. (2007). High-fidelity transmission of polarization encoded qubits from an entangled source over 100 km of fiber. *Optics Express*.

[3] Peng C., Zhang J., Yang D. (2007). Experimental long-distance decoy-state quantum key distribution based on polarization encoding. *Physical Review Letters*.

[4] Liao S.-K., Cai W.-Q., Liu W.-Y. (2017). Satellite-to-ground quantum key distribution. *Nature*.

[5] Mirhosseini M., Magaña-Loaiza O. S., O'Sullivan M. N. (2015). High-dimensional quantum cryptography with twisted light. *New Journal of Physics*.

[6] Sit A., Bouchard F., Fickler R. (2017). High-dimensional intracity quantum cryptography with structured photons. *Optica*.

[7] Mafu M., Dudley A., Goyal S. (2013). Higher-dimensional orbital-angular-momentum-based quantum key distribution with mutually unbiased bases. *Physical Review A*.

[8] Willner A. J., Ren Y., Xie G. (2015). Experimental demonstration of 20 Gbit/s data encoding and 2 ns channel hopping using orbital angular momentum modes. *Optics Letters*.

[9] Ren Y., Liu C., Pang K. (2017). Spatially multiplexed orbital-angular-momentum-encoded single photon and classical channels in a free-space optical communication link. *Optics Letters*.

[10] Erhard M., Fickler R., Krenn M., Zeilinger A. (2018). Twisted photons: new quantum perspectives in high dimensions. *Light: Science & Applications*.

[11] Mair A., Vaziri A., Weihs G., Zeilinger A. (2001). Entanglement of the orbital angular momentum states of photons. *Nature*.

[12] Wang X., Luo Y., Huang H. (2018). 18-Qubit entanglement with six photons’ three degrees of freedom. *Physical Review Letters*.

[13] Mirhosseini M., Malik M., Shi Z., Boyd R. W. (2013). Efficient separation of the orbital angular momentum eigenstates of light. *Nature Communications*.

[14] Pang K., Liu C., Xie G. (2018). Demonstration of a 10 Mbit/s quantum communication link by encoding data on two Laguerre-Gaussian modes with different radial indices. *Optics Letters*.

[15] Allen L., Beijersbergen M. W., Spreeuw R. J. C., Woerdman J. P. (1992). Orbital angular momentum of light and the transformation of Laguerre-Gaussian laser modes. *Physical Review A*.

[16] Yao A. M., Padgett M. J. (2011). Orbital angular momentum: origins, behavior and applications. *Advances in Optics and Photonics*.

[17] Liu C., Pang K., Song H. Experimental demonstration of a 20-Mbit/s per channel free-space bi-directional quantum communication link using orbital-angular-momentum encoding and multi-port mode converters.

[18] Ren Y., Huang H., Xie G. (2013). Atmospheric turbulence effects on the performance of a free space optical link employing orbital angular momentum multiplexing. *Optics Letters*.

[19] Anguita J. A., Neifeld M. A., Vasic B. V. (2008). Turbulence-induced channel crosstalk in an orbital angular momentum-multiplexed free-space optical link. *Applied Optics*.

[20] Krenn M., Handsteiner J., Fink M., Fickler R., Zeilinger A. (2015). Twisted photon entanglement through turbulent air across Vienna. *Proceedings of the National Acadamy of Sciences of the United States of America*.

[21] Jha A. K., Tyler G. A., Boyd R. W. (2010). Effects of atmospheric turbulence on the entanglement of spatial two-qubit states. *Physical Review A: Atomic, Molecular and Optical Physics*.

[22] Paterson C. (2005). Atmospheric turbulence and orbital angular momentum of single photons for optical communication. *Physical Review Letters*.

[23] Rodenburg B., Mirhosseini M., Malik M. (2014). Simulating thick atmospheric turbulence in the lab with application to orbital angular momentum communication. *New Journal of Physics*.

[24] Liu C., Pang K., Ren Y. Demonstration of adaptive optics compensation for emulated atmospheric turbulence in a two-orbital-angular-momentum encoded free-space quantum link at 10 Mbits/s.

[25] Ren Y., Xie G., Huang H. (2014). Adaptive-optics-based simultaneous pre- and post-turbulence compensation of multiple orbital-angular-momentum beams in a bidirectional free-space optical link. *Optica*.

[26] Willner A. E., Huang H., Yan Y. (2015). Optical communications using orbital angular momentum beams. *Advances in Optics and Photonics*.

[27] Bazhenov V., Vasnetsov M. V., Soskin M. S. (1990). Laser-beams with screw dislocations in their wave-fronts. *JETP Letters*.

[28] Heckenberg N. R., McDuff R., Smith C. P., White A. G. (1992). Generation of optical phase singularities by computer-generated holograms. *Optics Letters*.

[29] Wang J., Yang J.-Y., Fazal I. M. (2012). Terabit free-space data transmission employing orbital angular momentum multiplexing. *Nature Photonics*.

[30] Deng F.-G., Long G. L. (2004). Bidirectional quantum key distribution protocol with practical faint laser pulses. *Physical Review A: Atomic, Molecular and Optical Physics*.

[31] Shi G.-F., Xi X.-Q., Tian X.-L., Yue R.-H. (2009). Bidirectional quantum secure communication based on a shared private Bell state. *Optics Communications*.

[32] Parenti R. R., Roth J. M., Shapiro J. H., Walther F. G., Greco J. A. (2012). Experimental observations of channel reciprocity in single-mode free-space optical links. *Optics Express*.

[33] Chandrasekaran N., Shapiro J. H. (2014). Photon information efficient communication through atmospheric turbulence-part I: channel model and propagation statistics. *Journal of Lightwave Technology*.

[34] Labroille G., Denolle B., Jian P., Genevaux P., Treps N., Morizur J.-F. (2014). Efficient and mode selective spatial mode multiplexer based on multi-plane light conversion. *Optics Express*.

[35] Liu C., Pang K., Zhao J. Demonstration of single-end adaptive optics compensation for emulated turbulence in a Bi-directional 10-Mbits/s per channel free-space quantum communication link using orbital-angular-momentum encoding.

[36] Wang Q., Chen W., Xavier G. (2008). Experimental decoy-state quantum key distribution with a sub-poissionian heralded single-photon source. *Physical Review Letters*.

[37] Leonhard N., Sorelli G., Shatokhin V. N., Reinlein C., Buchleitner A. (2018). Protecting the entanglement of twisted photons by adaptive optics. *Physical Review A: Atomic, Molecular and Optical Physics*.

[38] Marcikic I., De Riedmatten H., Tittel W., Zbinden H., Legré M., Gisin N. (2004). Distribution of time-bin entangled qubits over 50 km of optical fiber. *Physical Review Letters*.

[39] Roddier F. (1988). Curvature sensing and compensation: a new concept in adaptive optics. *Applied Optics*.

[40] Ragazzoni R., Farinato J. (1999). Sensitivity of a pyramidic wave front sensor in closed loop adaptive optics. *Astronomy and Astrophysics*.

[41] Sandler D. G., Cuellar L., Lefebvre M. (1994). Shearing interferometry for laser-guide-star atmospheric correction at large *D/r*_0_. *Journal of the Optical Society of America A: Optics, Image Science & Vision*.

[42] Leonhard N., Berlich R., Minardi S. (2016). Real-time adaptive optics testbed to investigate point-ahead angle in pre-compensation of Earth-to-GEO optical communication. *Optics Express*.

[43] Lavery M. P. J. (2018). Vortex instability in turbulent free-space propagation. *New Journal of Physics*.

